# Co-Encapsulation of Curcumin and α-Tocopherol in Bicosome Systems: Physicochemical Properties and Biological Activity

**DOI:** 10.3390/pharmaceutics15071912

**Published:** 2023-07-09

**Authors:** Daniela Vergara, Olga López, Claudia Sanhueza, Catalina Chávez-Aravena, José Villagra, Mariela Bustamante, Francisca Acevedo

**Affiliations:** 1 Center of Excellence in Translational Medicine—Scientific Technological Bioresource Nucleus (CEMT-BIOREN), Faculty of Medicine, Universidad de La Frontera, Casilla 54-D, Temuco 4780000, Chile; claudiaandrea.sanhueza@ufrontera.cl (C.S.); francisca.acevedo@ufrontera.cl (F.A.); 2 Department of Chemical and Surfactant Technology, Institute of Advanced Chemistry of Catalonia (IQAC-CSIC), C/Jordi Girona 18-26, 08034 Barcelona, Spain; olga.lopez@iqac.csic.es; 3 Laboratory of Pharmaceutical and Cosmetic Bioproducts, Center of Excellence in Translational Medicine (CEMT), Department of Preclinical Sciences, Faculty of Medicine, Universidad de La Frontera, Casilla 54-D, Temuco 4780000, Chile; catalina.chavez@ufrontera.cl (C.C.-A.); jose.villagra@ufrontera.cl (J.V.); 4 Center of Food Biotechnology and Bioseparations, Scientific and Technological Bioresource Nucleus BIOREN, Universidad de La Frontera, Casilla 54-D, Temuco 4780000, Chile; mariela.bustamante@ufrontera.cl; 5 Department of Basic Sciences, Faculty of Medicine, Universidad de La Frontera, Casilla 54-D, Temuco 4780000, Chile

**Keywords:** curcumin, α-tocopherol, bicosomes, delivery system, skin, oxidative stress, candidiasis

## Abstract

A novel co-encapsulation system called bicosomes (bicelles within liposomes) has been developed to overcome the limitations associated with the topical application of curcumin (cur) and α-tocopherol (α-toc). The physicochemical properties and biological activity in vitro of bicosome systems were evaluated. Bicelles were prepared with DPPC, DHPC, cur, and α-toc (cur/α-toc-bicelles). Liposomal vesicles loading cur/α-toc-bicelles were prepared with Lipoid P-100 and cholesterol-forming cur/α-toc-bicosomes. Three cur/α-toc-bicosomes were evaluated using different total lipid percentages (12, 16, and 20% *w*/*v*). The results indicated that formulations manage to solubilize cur and α-toc in homogeneous bicelles < 20 nm, while the bicosomes reaches 303–420 nm depending on the total lipid percentage in the systems. Bicosomes demonstrated high-encapsulation efficiency (EE) for cur (56–77%) and α-toc (51–65%). The loading capacity (LC) for both antioxidant compounds was 52–67%. In addition, cur/α-toc-bicosomes decreased the lipid oxidation by 52% and increased the antioxidant activity by 60% compared to unloaded bicosomes. The cell viability of these cur/α-toc-bicosomes was >85% in fibroblasts (3T3L1/CL-173™) and ≥65% in keratinocytes (Ha-CaT) and proved to be hematologically compatible. The cur/α-toc-bicelles and cur/α-toc-bicosomes inhibited the growth of *C. albicans* in a range between 33 and 76%. Our results propose bicosome systems as a novel carrier able to co-encapsulate, solubilize, protect, and improve the delivery performance of antioxidant molecules. The relevance of these findings is based on the synergistic antioxidant effect of its components, its biocompatibility, and its efficacy for dermal tissue treatment damaged by oxidative stress or by the presence of *C. albicans*. However, further studies are needed to assess the efficacy and safety of cur/α-toc bicosomes in vitro and in vivo.

## 1. Introduction

In recent years, antioxidant compounds have attracted the attention of researchers due to their diverse dermatological, pharmaceutical, and biological functions. These compounds exhibit characteristics such as free radical scavenging, melanin reduction, wound healing, and light protection. [1,2]. Curcumin (cur) is a hydrophobic polyphenol extracted from the rhizome of *Curcuma longa* [3]. Several studies have demonstrated the biological activity of cur, which includes antioxidant, anti-inflammatory, antimicrobial, anticancer, and wound-healing effects [4]. On the other hand, α-tocopherol (α-toc) is the most active and effective form of vitamin E, which is widely distributed in nature and used as an antioxidant in the food, cosmetics, and pharmaceutical industries [5,6,7]. In addition, the topical application of α-toc has been proven to have a protective effect on the skin and accelerate wound closure [8]. Furthermore, due to its peroxyl radical-scavenging activity, α-toc can help protect the phospholipids and fatty acids in the skin’s membrane [1]. Despite the remarkable effectiveness of cur and α-toc in dermatological applications, its therapeutic value is limited by several factors. Cur exhibits poor aqueous solubility (<0.1 mg/mL), low physicochemical stability, unsustainable bioavailability, low skin permeability and fast metabolism that contribute to low cur levels in tissues [9,10,11,12]. On the other hand, α-toc is easily oxidized by atmospheric oxygen, shows low solubility in water (~30 mg/L) and high sensitivity to light, and some skin irritation issues have been described limiting its application and incorporation in products [13,14]. These challenges represent significant obstacles to achieving optimal topical and systemic efficacy of cur and α-toc. To overcome these drawbacks, the development of co-encapsulation delivery systems offers several advantages, including the protection of therapeutic molecules and integrity, facilitation of incorporation, formulation flexibility in terms of ratio and dosage, controlled release capabilities, and the potential for synergistic effects when multiple therapeutic molecules are incorporated [15,16]. The construction of innovative co-encapsulation administration systems is crucial for the advancement of new products for dermatological applications [17].

Bicosome systems are phospholipid assemblies based on mixtures of discoidal structures called bicelles and spherical vesicles called liposomes. Studies propose the use of phospholipid-based bicelles for dermatological applications owing to their lipid composition and their small size (15–25 nm in diameter and 4–6 nm thick), suitable enough for passing through the skin (*stratum corneum*) [18]. However, in high-dilution conditions, small discoidal bicelles become large structures such as vesicles, lamellar sheets, and rodlike micelles losing their specific properties. To maintain the size and shape of the small discoidal bicelles, Rodriguez et al. [19] suggested that bicelles can be encapsulated in larger spherical vesicles liposomes. These liposomal vesicles, with diameters ranging from 100 nm to 1 mm, are too large to pass through the skin for transdermal application; however, they are morphologically stable under high-dilution conditions and thus are good carriers for the systemic application of therapeutic molecules. When bicosome structures contact the skin surface, the external vesicle (liposome) fuses on the skin surface, while the discoidal bicelles selectively penetrate the lipid inter-corneocyte spaces to reach the target skin layers [20].

Previous studies on the application of these systems have shown that treatment with bicosomes containing ceramides improves the dermatitis skin condition in vivo by reinforcing the skin barrier function and reducing the skin inflammation [21]. Furthermore, bicosome systems have demonstrated their ability to effectively incorporate lipophilic and hydrophilic antioxidants [22]. Fernández et al. [23] evaluated the antioxidant protective effect of bicosomes that incorporate β-carotene against infrared radiation by measuring the formation of free radicals. Results indicated that treatment with β-carotene-loaded bicosomes reduces free radical formation, preserves collagen structure, and protects antioxidant β-carotene against radiation, demonstrating the potent efficacy of bicosomes in protecting skin exposed to infrared radiation [24,25].

Considering the important effects of cur and α-toc on oxidative processes and skin damage repair and the advantages of bicosomes as carrier and delivery systems [26,27], the co-encapsulation of cur and α-toc into bicosomes (cur/α-toc-bicosomes) is proposed. To the best of our knowledge, liposomal vesicles have been successfully used to load cur and α-toc, respectively [11,28]; however, there has been no reference to simultaneous encapsulation in a bicosome co-delivery system as described above. The aims of the present study were to develop and characterize the physicochemical properties and biological activity in vitro of cur/α-toc-bicosomes. The influence of the concentrations of phospholipids and cholesterol in three cur/α-toc-bicosomes was studied. The cur/α-toc-bicosomes were characterized in terms of particle size, polydispersity index (PDI), morphology, Fourier-transform infrared (FTIR), encapsulation efficiency (EE), loading capacity (LC), and lipid oxidation. Finally, the cur/α-toc-bicosomes were evaluated according to antioxidant, hemolytic, cell viability, and antifungal activity. This study provides a promising strategy to improve the solubility and the antioxidant effectiveness of cur and α-toc through a novel co-encapsulation skin delivery system. The proposed systems could be used for the topical treatment of diseases caused by oxidative damage or as dermal antifungal therapy.

## 2. Materials and Methods

### 2.1. Chemicals

1,2-di-palmitoyl-sn-glycero-3-phosphocholine (DPPC) and 1,2-dihexanoyl-sn-glycero-3-phosphocholine (DHPC) were purchased from Avanti Polar Lipids (Alabaster, AL, USA). Lipoid P-100 phosphatidylcholine (>97%) from soybean non-(GMO) was kindly supplied by Lipoid GmbH (Ludwigshafen, Germany). Cholesterol, curcumin (cur ≥ 65%), α-tocopherol (α-toc ≥ 96%), malondialdehyde (MDA), and 1,1,3,3-tetraethoxypropane (TEP) were obtained from Sigma-Aldrich (St. Louis, MO, USA). Purified water was obtained from an ultrapure water system (Thermo Scientific Barnstead MicroPure ST, Langenselbold, Germany). HPLC grade chloroform, HPLC grade acetonitrile, HPLC grade methanol, 3-(4,5-dimethylthiazol-2-yl)-2,5-diphenyl tetrazolium bromide (MTT), 1, 1 diphenyl 2-picryl-hydrazyl (DPPH), thiobarbituric acid (TBA), and Triton X-100 were purchased from Merck. Trolox was obtained from Santa Cruz Biotechnology Inc. (Dallas, TX, USA). Dulbecco’s Modified Eagle Medium (DMEM), DMEM without phenol red, fetal bovine serum, penicillin, streptomycin, fetal bovine serum (FBS), and Trypsin-EDTA were purchased from Thermo Fisher Scientific (Waltham, MA, USA). Peptone, Yeast Extract–Peptone–Dextrose Broth, and Sabouraud Dextrose Agar were obtained from Becton, Dickinson and Company (Sparks, MD, USA).

### 2.2. Preparation of the Systems

#### 2.2.1. Bicelles

Bicellar formulations were prepared by the thin-layer dispersion method, and concentrations were determined from preliminary experiments. Briefly, bicelles were formed with DPPC/DHPC at a 3.5:1 lipid molar ratio (*q*), cur (180 µM), and α-toc (600 µM). The mixture was dissolved in chloroform into a round-bottom flask. The chloroform was removed with a rotary evaporator (Büchi Rotavapor R-100, Flawil, Switzerland) at 40 °C; thin lipid films were formed on the flask walls. The dried lipid films were hydrated with distilled water to reach 6% *w*/*v* of total lipid concentration. Bicellar formulations were prepared by subjecting the sample to several cycles of sonication and freezing until the sample became transparent. Cur and α-toc-loaded bicelles were called cur/α-toc-bicelles.

Different studies related to bicelles [29] and our own experience [19,20] indicate that DPPC/DHPC at *q* = 3.5:1 discoidal system is obtained easily and they remain stable. In addition, the total concentration of 6% is adequate to maintain the discoidal morphology of the bicelles, since lower concentration could induce the transformation of bicelles to vesicles [20].

#### 2.2.2. Bicosome Systems

The liposomal vesicles were prepared using Lipoid P-100/cholesterol in a ratio of 8:2 at different concentrations 6, 10, and 14% *w*/*v*. These concentrations were chosen to achieve the encapsulation of the bicelles while maintaining the fluidity of the final systems. Each mixture was dissolved in chloroform into a round-bottom flask. The chloroform was removed with a rotary evaporator at 40 °C. The film was hydrated with the cur/α-toc-bicelles (6% *v*/*w*) until bicosomes formed; these bicosomes were called cur/α-toc-bicosomes. Three cur/α-toc-bicosomes were prepared (B_A_, B_B_, and B_C_), reaching 12, 16 and 20% of total lipids, respectively. In general, the lipid concentration was high to favor the incorporation of the two studied molecules, cur and α-toc. Control bicosomes were formed according to the same methods, without cur and α-toc. The cur/α-toc-bicosomes represent a novel encapsulation method that combines the advantages of both bicelles and liposomes. This approach aims to enhance solubility, increase entrapment efficiency, reduce lipid oxidation, and improve antioxidant activity. Table 1 summarizes the components, concentrations, and percentage of mass (%) used for each formulation.

### 2.3. Characterization of the Systems

The characterization was addressed to evaluate the size and morphology of the studied systems (Dynamic Light Scattering and microscopy) and help us to understand how the inclusion of the studied antioxidants affects bicelles and bicosomes. In addition, FTIR spectroscopy suggested how antioxidants interact with lipids. Encapsulation efficiency (EE) and loading capacity (LC) reported on the ability of the systems as carriers of the molecules studied. These techniques are commonly employed in characterization studies of similar systems, enabling us to effectively compare our results with those of other studies.

#### 2.3.1. Determination of Particle Size and Polydispersity Index (PDI)

The particle size and PDI of the bicellar formulations and the cur/α-toc-bicosomes (B_A_, B_B_, and B_C_) were determined using a Zetasizer Nano ZS (HT series, Malvern Instruments, Malvern, UK) at 25 °C. Conditions for measurement were defined according to Liu et al. [30]. The relative refractive index, i.e., the ratio of the refractive index of the phospholipids (1.490) to that of the dispersion medium (1.330), was 1.120. The absorption of the phospholipids was 0.001.

#### 2.3.2. Morphology

The morphology of the cur/α-toc-bicosomes was observed using confocal laser scanning microscopy (CLSM, Zeiss LSM780, Jena, Germany) and scanning transmission electron microscopy (STEM, Hitachi SU-3500, Tokyo, Japan). The sample (1 mL) was mixed with 40 μL of BODIPY^®^ (Thermo Fisher Scientific, Molecular Probes™) to stain the phospholipids. The mixed solution was placed on a concave confocal microscope slide. Images of the sample were acquired with a 100× magnification lens. For the image acquisition in STEM, the cur/α-toc-bicosomes (1 mL) were incubated in 1% osmium tetroxide solution (Sigma-Aldrich) for 2 h at 4 °C. The excess of the solution was removed by centrifugation (5000× *g* by 10 min).

#### 2.3.3. Fourier-Transform Infrared (FTIR) Spectroscopy

The chemical structure of lyophilized cur/α-toc-bicosomes, free cur, and free α-toc was monitored by FTIR spectroscopy using the Jasco FT-IR-4600 spectrophotometer (Jasco Corporation, Tokyo, Japan) equipped with an attenuated total reflection (ATR) accessory that uses a ZnSe crystal at an angle of incidence of 45° on a horizontal orientation. The spectra were collected in the wavenumber between 500 and 4500 cm^−1^. Scan speed was 20 scan/sec at a resolution of 16 cm^−1^ at 25 °C. Peaks obtained were identified, compared, and interpreted from reference spectra.

#### 2.3.4. Determination of Encapsulation Efficiency (EE) and Loading Capacity (LC)

To determine the percentage of cur/α-toc-bicosomes (B_A_, B_B_, and B_C_), 40 µL of each system was centrifuged at 12,000× *g* for 30 min at 20 °C (Centurion Scientific Limited K2015R, Chichester, UK). The supernatant was discarded, and the precipitate was diluted in a mixture of acetonitrile, methanol, and water (88/8/4 *v*/*v*/*v*). The concentrations of cur and α-toc were determined by a reverse-phase HPLC system (Jasco, Japan) equipped with a quaternary pump (PU-4180), an auto-injector (AS-4150), a UV–vis photodiode array detector (MD-4010), a column C18 Inertsil^®^ ODS-4 (5 µm × 4.6 mm × 250 mm), and Chromnav data software 2.02.08. The components of the mobile phase were pumped into the chromatographic system at a flow rate of 1.4 mL/min in gradient mode: at 0 min 47.5% (*v*/*v*) acetonitrile, 47.5% (*v*/*v*) water, and 5% (*v*/*v*) methanol, between a 9 and 12 min transition to 50% (*v*/*v*) acetonitrile and 50% (*v*/*v*) methanol. At 12–23 min 50% (*v*/*v*) acetonitrile and 50% (*v*/*v*) methanol; at a 23–25 min transition to 47.5% (*v*/*v*) acetonitrile, 47.5% (*v*/*v*) water, and 5% (*v*/*v*) methanol; at 25–30 min, to 47.5% (*v*/*v*) acetonitrile, 47.5% (*v*/*v*) water, and 5% (*v*/*v*) methanol. Aliquots of 20 μL were analyzed at 425 and 292 nm to determine the cur and α-toc, respectively. The cur and α-toc peaks were obtained based on their retention time (9.6 and 22.5 min, respectively). Finally, cur and α-toc concentrations were calculated from standard curves obtained by reading the values of absorbance of solutions containing cur (0.5–50 ppm) with a R^2^ of 0.999 and α-toc (5–100 ppm) with a correlation coefficient (R^2^) of 0.999. The *EE* was determined using the following Equation (1):

(1) $$EE\;\left(\%\right)\;=\;\frac{Loaded\;cur\;or\;\alpha -toc\;content}{Initial\;amount}\times 100$$

*LC* was calculated as the amount of cur and α-toc entrapped in the bicosomes (B_A_, B_B_, and B_C_) versus the total amount of lipids used (*cur*, *α-toc*, DHPC, DPPC, Lipoid P-100, and cholesterol) in the bicosomes preparation, according to the following Equation (2):

(2) $$LC\;\left(\%\right)\;=\;\frac{Encapsulated\;amount\;cur\;and\;\alpha -toc}{Total\;amount\;of\;lipids\;used}\;\times 100$$

The initial amount of *cur*, *α-toc*, and total lipids used, and the encapsulated amount of cur and α-toc were calculated in mg/mL.

### 2.4. Lipid Oxidation

The TBA reactive substances (TBARS) method was used following the methodology described by Vergara and Shene [31]. Briefly, a solution of trichloroacetic acid (TCA)–TBA–HCl was prepared by mixing 15 g TCA, 375 mg TBA, 1.76 mL 12 N HCl, and 82.9 mL H_2_O. One mL of the TCA–TBA–HCl solution was mixed with 200 μL of each cur/α-toc-bicosomes (B_A_, B_B_, and B_C_) and unloaded bicosomes. The mixture was incubated at 95 °C for 30 min; after being cooled down to room temperature, the mixture was centrifuged at 4000× *g* for 5 min, and the absorbance was measured at 531 nm. Results were expressed as μg of MDA equivalent per mg of sample, based on a TEP standard curve, in a concentration range between 0.0 and 3.6 µg/mL.

### 2.5. Antioxidant Activity

The free radical scavenging capacity of cur/α-toc-bicosomes (B_A_, B_B_, and B_C_) and unloaded bicosomes was measured by the DPPH assay. Briefly, 10 µL of each bicosome were mixed with 290 µL of ethanol solution of DPPH at 150 µM and incubated for 30 min at 25 °C in the absence of light. Ethanol was used as a negative control. The absorbance of the reaction solution was measured at 517 nm in a microplate reader (SPECTROstar Nano, BMG LABTECH, Cary, NC, USA). The percentage of DPPH radical scavenging by the sample was calculated according to Equation (3):

(3) $$Scavenging\;activity\;\left(\%\right)\;=\;\frac{A_{0}\;-\;A_{1}}{A_{0}}\;\times 100$$

where *A*_0_ is the absorbance of the control (blank, without sample) and *A*_1_ is the absorbance in the presence of the sample formulation.

### 2.6. Hemolytic Activity

Fresh human whole blood from anonymized healthy donors was obtained from the Center of Excellence in Translational Medicine, Universidad de La Frontera (Temuco, Chile). Experiments were conducted under approval of Universidad de La Frontera Scientific Ethics Committee (protocol No. 099-20). Blood was diluted 1:30 in 0.9% NaCl solution. The cur/α-toc-bicosomes (B_A_, B_B_, and B_C_) (100 μL) were individually immersed in Eppendorf tubes containing 1 mL of the blood solution. At the same time, saline solution (100 μL) and Triton X-100 4% (*v*/*v*) (100 μL) were used as the negative and positive control, respectively. The experiments were carried out in triplicate. The tubes were placed on an orbital shaker at 100 rpm and maintained at 37 °C. After 60 min of incubation, 1 mL of the suspension was removed from each tube and centrifuged for 10 min at 10,000× *g*. The absorbance of the supernatant (100 μL) was measured at 540 nm using a microplate reader (SPECTROstar Nano, BMG LABTECH). Percentages of hemolysis were calculated using Equation (4):

(4) $$Hemolysis\;\left(\%\right)\;=\;\frac{Abs\;-\;{Abs}_{nc}}{{Abs}_{pc}\;-\;{Abs}_{nc}}\;\times 100$$

where *Abs* corresponds to the bicosomes absorbance, *Abs_pc_* corresponds to the positive control absorbance, and *Abs_nc_* to the negative control absorbance.

### 2.7. Cell Culture

Dermal fibroblast (3T3L1/CL-173™) and human keratinocyte (HaCaT) cell lines were obtained from the American Type Culture Collection (ATCC; Manassas, VA, USA). Both cell lines were gently provided by the Laboratory of Integrative Biology, Universidad de La Frontera. The cells were cultured in DMEM supplemented with 10% (*v*/*v*) FBS and 1% (*v*/*v*) penicillin and streptomycin. Cells were incubated at 37 °C in a 95% humidified atmosphere and 5% CO_2_. Flasks were sub-cultured when they were 80–90% confluent. Three-minute exposure to 0.25% *w*/*v* trypsin-EDTA (Corning, Somerville, MA, USA) was used to release attached cells from the tissue culture surface.

#### Biocompatibility and Cell Viability

The cell viability keratinocyte (HaCaT) and fibroblast (3T3L1/CL-173™) following treatment with different concentrations of cur/α-toc-bicosomes and free cur and α-toc solution was evaluated by MTT assay.

Free cur and α-toc solutions were prepared in DMSO. Cells were seeded at a density of 1 × 10^4^ cells per well with a final volume of 200 μL/well. Cells were treated with either a cur/α-toc-bicosome (B_A_, B_B_, and B_C_) or free cur and α-toc solutions at different concentrations (0.5–25 μM). Next, the cells were incubated for 24 h and kept in 5% CO_2_ conditions at 37 °C. After this period, the treatments were removed and (200 μL) MTT solution was added per well and incubated for 2 h at 37 °C. Then, the MTT solution was removed, and isopropanol (150 μL) was added to each well to dissolve formazan crystals and the plates were shaken for 5 min. Absorbance was recorded using a microplate reader (SPECTROstar Nano, BMG LABTECH), at 570 nm for the test, blank, and control wells. Percentage cell viability was calculated using the following Equation (5):

(5) $$Cell\;viability\;\left(\%\right)\;=\;\frac{{Abs}_{\left(sample\right)}\;-\;{Abs}_{\left(blank\right)}}{{Abs}_{\left(control\right)}\;-\;{Abs}_{\left(blank\right)}}\;\times 100$$

where *Abs* represents the absorbance values of the wells with test samples, buffer medium, and untreated cells. For each experiment, the absorbance was the average value measured in a microplate reader.

### 2.8. Antifungal Activity

To determine in vitro antifungal activity, the selected fungal strain *Candida albicans* was obtained from the Bioprocesses and Bioseparations Laboratory (Universidad de La Frontera, Temuco, Chile). The antifungal effect of cur/α-toc-bicelles and cur/α-toc-bicosomes (B_A_, B_B_, and B_C_) on *C. albicans* was determined by the standard plate count method. Briefly, the inoculum of *C. albicans* was cultured in Yeast Extract–Peptone–Dextrose broth for 18 h at 37 °C and was used at a turbidity of 1 × 10^8^ colony forming units (CFU/mL) according to the MacFarland scale. The inoculum was mixed with cur/α-toc-bicelles and cur/α-toc-bicosomes (B_A_, B_B_, and B_C_) at 50:50 (inoculum:formulation) ratio. The control was prepared by replacing the proportion of formulation with sterile milliQ water; samples were incubated for 24 h at 37 °C. Subsequently, the samples were subjected to serial dilutions in sterile buffered peptone water (4.9 mL; 0.1% w/v); the appropriate dilution of the cell suspension was seeded on Sabouraud Dextrose Agar for 48 h at 37 °C. Then, cell counting was performed. Cell viability during storage was expressed as colony forming units per mL (CFU/mL). Percentage inhibition was calculated using the following Equation (6):

(6) $$Inhibition\;\left(\%\right)\;=\;100-\left(\frac{N-100}{N_{0}}\right)$$

where *N* represents CFU/mL of the formulation sample and *N*_0_ represents CFU/mL of the control sample.

### 2.9. Statistical Analysis

Statistical analyses were performed using Graph-Pad Prism 9.0 software. Data from three independent experiments were expressed as means ± standard deviations (SD). Statistical significance was evaluated based on one-way ANOVA followed by tukey’s test (particle size, volume, PDI, EE, LC, and antifungal effect) and two-way ANOVA followed by Bonferroni’s or Dunnett´s method after validating the normality of the data set (MDA, DPPH, and cell viability). A confidence level of α < 0.05 was considered statistically significant.

## 3. Results and Discussion

### 3.1. Characterization of Bicelles and Bicosomes

#### 3.1.1. Particle Size and Polydispersity Index (PDI)

The hydrodynamic diameter and polydispersity index (PDI) of unloaded bicelles, cur/α-toc-bicelles, and cur/α-toc-bicosomes (B_A_, B_B_, and B_C_) were determined using dynamic light scattering. Typically, particle size analysis is performed based on the intensity (%) of scattered light. However, in the case of bicelles, their small size, discoidal shape, and particle motion pose challenges to accurate measurements. Moreover, in heterogeneous systems such as bicosomes (composed of bicelles and liposomes), larger and smaller particles contribute differently to the intensity of scattered light. Consequently, analyzing by intensity (%) can lead to an inflated proportion of larger particles. To address these limitations, we analyzed our results by volume (%) instead. The results are presented in Table 2 and Table 3, providing a more accurate representation of the particle size in our study. Particle size by intensity (%) for cur/α-toc-bicosomes (B_A_, B_B_, and B_C_) is shown in Supplementary Table S1.

**Figure 1:**
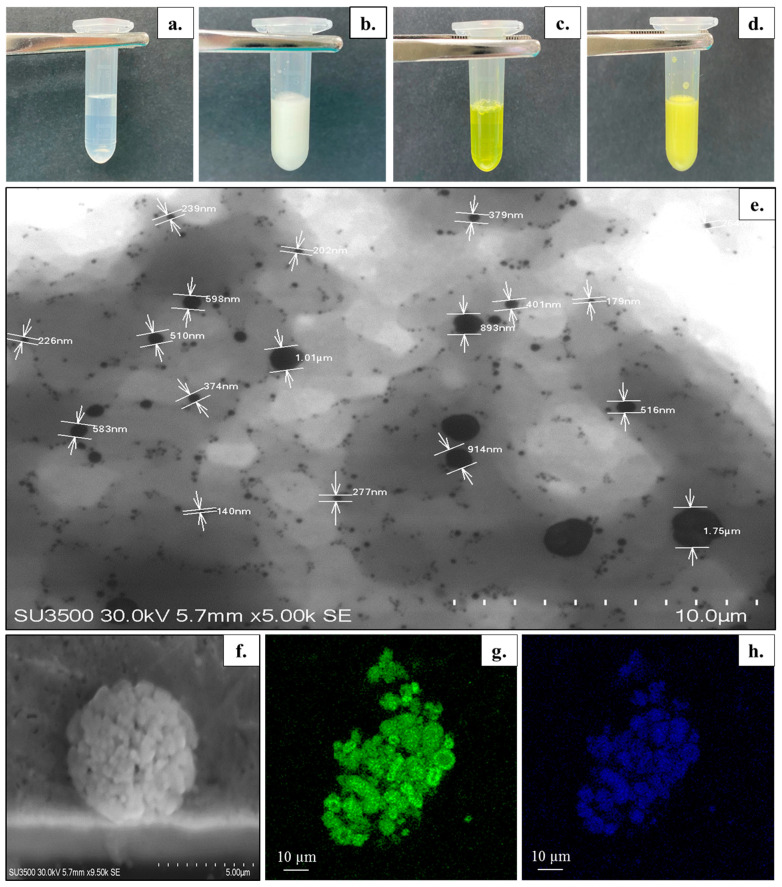
(**a**–**d**) Visual appearance of prepared formulations: (**a**) unloaded bicelles, (**b**) unloaded bicosomes, (**c**) cur/α-toc-bicelles, and (**d**) cur/α-toc-bicosomes. (**e**–**h**) Morphology of cur/α-toc-bicosomes. (**e**–**f**) Scanning transmission electron microscopy images. (**g**,**h**) Confocal laser scanning microscopy images. (**g**) Staining of the phospholipid membrane of cur/α-toc-bicosomes by BODIPY**^®^** (green color), (**h**) Auto-fluorescence of cur (blue color).

The particle sizes of unloaded bicelles and cur/α-toc-bicelles did not exhibit any significant differences (*p*-value < 0.05), measuring at approximately 16 ± 1 nm and 15 ± 0.1 nm, respectively. This indicates that the inclusion of cur and α-toc did not affect the size of the bicelles. The PDI value, which represents the uniformity of particle size, is calculated as the ratio of the standard deviation to the mean particle size. A PDI value around 0.10 suggests monodispersity, values between 0.10 and 0.40 indicate a narrow particle size distribution, while values close to 1.0 suggest less uniformity in particle size [32]. For unloaded bicelles, the PDI value was measured at 0.26 ± 0.02, and for cur/α-toc-bicelles, it was found to be 0.28 ± 0.07, indicating a narrow particle size distribution. The homogeneity of the bicellar formulations can be observed in Figure 1a,c. The small average particle size and the homogeneity of cur/α-toc-bicelles are advantageous characteristics for the stability, solubility, and bioavailability of the formulations [33].

On the other hand, the particle size of cur/α-toc-bicosomes (B_A_, B_B_, and B_C_) presents a bimodal size distribution or two main peaks (Table 3) (Figure S1). Peak 1 exhibited particle sizes ranging from 31 to 60 nm, while peak 2 showed particle sizes ranging from 303 to 420 nm. The analysis by volume (%) revealed a higher proportion (≥57%) of small particles (≤60 nm) than large ones, indicating the predominant presence of small vesicles mainly in B_B_ and B_C_. The particle sizes in peak 1 increased with increasing concentration of phosphatidylcholine (Lipoid P-100) and cholesterol. This could be due to an increase in the concentration of these lipids, promoting the formation of liposomal vesicles; therefore, more bicelles can be encapsulated in them. A lower number of bicelles outside the liposomal vesicle means the average particle size of peak 1 increases. Therefore, a particle size of 55–60 nm would correspond to a higher proportion of liposomal vesicles than bicelles. In addition, cur/α-toc-bicelles not encapsulated in liposomal vesicles could be transformed from disks to vesicles in the bicosome formation process. Rodriguez et al. [20] reported that dilution of bicosomes promoted the growth of structures, inducing the increase in particle size average. This could explain the behavior of peak 2, which increases in size when the concentration of lipids forming the liposomal vesicle decreases. The bimodal distribution of the bicosomes increases the PDI until it reaches a value of 1.0 as expected. Changes in the physical stability of cur/α-toc-bicosome (B_C_ formulation) were determined by particle size (nm) initially and after 90 days storage at 4 °C (Supplementary Figure S2). The results showed no significant changes in particle size.

#### 3.1.2. Visual Appearance and Microscopic Structure

The visual appearance of the unloaded bicelles, cur/α-toc-bicelles, unloaded bicosomes, and cur/α-toc-bicosomes is shown in Figure 1a–d. The unloaded bicelles and cur/α-toc-bicelles turn from colorless to yellow, acquiring the characteristic coloration of cur (Figure 1a,c). Additionally, the transparency in cur/α-toc-bicelles indicates that these are able to incorporate and solubilize both insoluble molecules in large amounts of water. The unloaded bicosomes and cur/α-toc-bicosomes correspond to formulations with a milky appearance (Figure 1b,d). In addition, no agglomeration, precipitation, or phase separation was observed in any of the systems prepared (bicelles and bicosomes). STEM and CLSM were used to provide further information about the size and morphology of the cur/α-toc-bicosomes (Figure 1e–h). The bicosomes correspond to the spherical vesicles distributed throughout the image (Figure 1e). Two distributions of the particle size were observed vesicles around 200–400 nm and a large number of nano-sized vesicles <100 nm which is highly consistent with the results of the average particle size measured by the dynamic light scattering system, which showed two main peaks in the formulations (Table 3). In addition, larger particles (>500 nm) originate from the aggregation or adhesion of vesicles, as can be seen in Figure 1f. Figure 1g shows the phospholipid bilayer (green), due to fluorescent dye BODIPY^®^, whereas Figure 1h shows the auto-fluorescence of cur (blue), which is located in the same places where the phospholipid bilayer is found, indicating that cur is within the phospholipid bilayer.

#### 3.1.3. Fourier-Transform Infrared Spectroscopy (FTIR)

**Figure 2:**
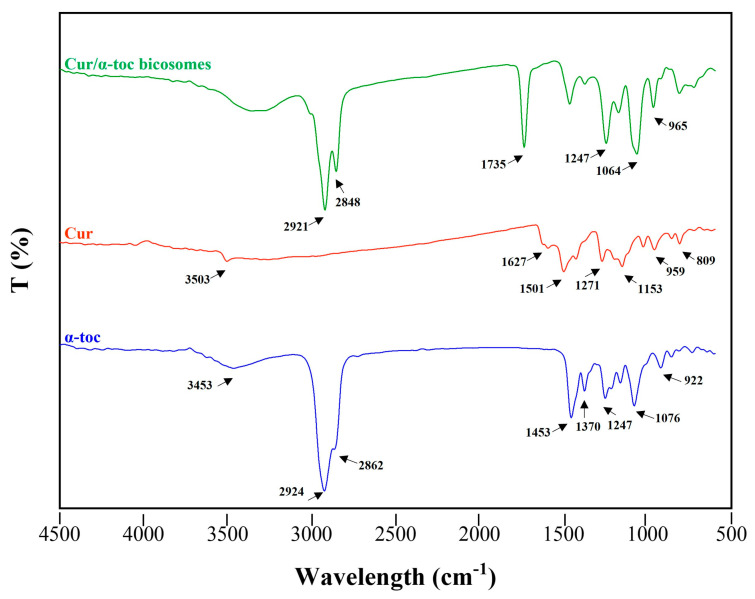
Fourier-transform infrared (FTIR) spectrum of bicosome systems and antioxidant compounds used in the formulation. (─) cur/α-toc-bicosomes, (─) free cur, and (─) free α-toc.

FTIR spectroscopy was used to study the interaction between cur, α-toc, and the bicosomes (Figure 2). The characteristic peaks of cur can be found at 3503 cm^−1^, a representative peak of the phenolic O–H group stretching vibrations. The peaks at 1627 and 1501 cm^−1^ are associated with the C=O and vibration of the benzene ring stretching respectively. The absorption peaks at 1271 and 1153 cm^−1^ are associated with aromatic C=O stretching and C–O–C group. The region between 959 and 809 cm^−1^ showed the cis and trans C–H deformation vibration on the benzene ring. In the cur spectrum, there were no peaks in the most significant carbonyl region (1800–1650 cm^−1^), indicating that cur existed in the keto-enol tautomeric form [34].

On the other hand, α-toc exhibited characteristic peaks at 3453 cm^−1^ (stretching –OH), 2924, and 2862 cm^−1^ (asymmetric and symmetric stretching vibrations of –CH_2_ and –CH_3_). The peak at 1453 cm^−1^ coincides with phenyl skeletal or methyl asymmetric bending, while 1370 cm^−1^ corresponds to methyl symmetric bending, 1247 cm^−1^ for –CH_2_ stretching bending, and 1076 cm^−1^ for plane bending of phenyl and 922 cm^−1^ for trans =CH_2_ stretching [35].

The cur/α-toc-bicosomes exhibited combined peaks of the phospholipids and cholesterol. The two absorption peaks at 2921 and 2848 cm^−1^ are attributed to the symmetric and asymmetric stretching vibration of the C–H bond in CH_2_, respectively. The peak at 1735 cm^−1^ represents the symmetric stretching vibration C=O group. In addition, the peaks at 1064 and 1247 cm^−1^ are related to the symmetric and asymmetric stretching vibration of PO^2−^. The peak at 965 cm^−1^ corresponds to the asymmetric stretching vibration of N(CH_3_)^3+^ [36]. However, most of the characteristic peaks of cur and α-toc could not be found in the cur/α-toc-bicosomes. This result demonstrated that the characteristic peaks of cur and α-toc merged or overlapped with the absorption bands of the lipid matrix. The above could occur because the lipid concentration used to prepare the bicosomes is higher than the concentration of cur/α-toc-loaded in them. However, Ng et al. [37] established the successful encapsulation of bioactive compounds of interest when the characteristic bands are not distinguishable in the complete encapsulation system.

#### 3.1.4. Encapsulation Efficiency (EE) and Loading Capacity (LC)

Two important physicochemical parameters in the optimization of bicosomes are the EE and LC. The EE of the bicosomes represents the fraction of added cur and α-toc that ends up in the bicelles and liposomal vesicles. A high EE ensures the bioavailability of cur and α-toc. The contents of cur and α-toc encapsulated in the bicosomes (B_A_, B_B_, and B_C_) were measured as shown in Table 4. The interesting results of EE may occur for several reasons. The high hydrophobicity of the phospholipids was used to prepare the bicosomes, since cur and α-toc are stabilized within the hydrophobic region of the phospholipids. In the bicosomes, both cur and α-toc have a double possibility of encapsulation; for example, the antioxidant compounds that were not encapsulated in the phospholipid membrane of the bicelles can then be encapsulated in the phospholipid membrane of the liposomal vesicle. On the other hand, by increasing the lipid percentage in the liposomal composition, more liposomal vesicles can be formed, increasing the encapsulation of cur/α-toc-bicelles and the EE of the bicosomes. Additionally, the increase in the lipid percentage in the liposomal composition may be directly related to the average vesicle size in the bicosomes (Table 3), as larger amounts of cur/α-toc-bicelles may be incorporated into larger liposomal vesicles possessing a larger internal diameter. In this case, B_C_ > B_B_ > B_A_ reached 77 ± 2%, 69 ± 4%, and 56 ± 5% of EE for cur, while for α-toc, the EE obtained was B_C_ > B_B_ > B_A_ reaching 65 ± 3%, 64 ± 2%, and 58 ± 8%, respectively.

Cur and α-toc incorporated in the formulations are hydrophobic compounds with large molecular structures and compete for space in the hydrophobic region of the bicelles and liposomal vesicles. The differences in the EE between cur and α-toc could be attributed to differences in the molecular structure of both antioxidant compounds. Cur could be incorporated into the hydrophobic region more effectively because it has a lower molecular weight (368 g/mol) than α-toc (431 g/mol).

In general, the EE reported for cur and α-toc in lipid systems is diverse and influenced by the nature of the encapsulating material, chemical properties, methodology used, and other reasons [38]. In addition, cur and α-toc are labile compounds; conditions such as light, temperature, and processing may also influence the result. The EE values in lipid systems have been reported in the range from 60% to 95% [39,40] for cur and from 10% to 90% for α-toc [5,41]. Thus, our results are in a highly acceptable range.

The LC of the bicosomes corresponds to the fraction of the bicelles and liposomal vesicles comprised of cur and α-toc. The LC of cur/α-toc-bicosomes was analyzed as shown in Table 4. The highest LC was obtained by the B_C_ formulation (67 ± 2%) followed by B_B_ and B_A_ formulations (65 ± 2% and 52 ± 0.4%, respectively), which is consistent with the EE results.

### 3.2. Lipid Oxidation

**Figure 3:**
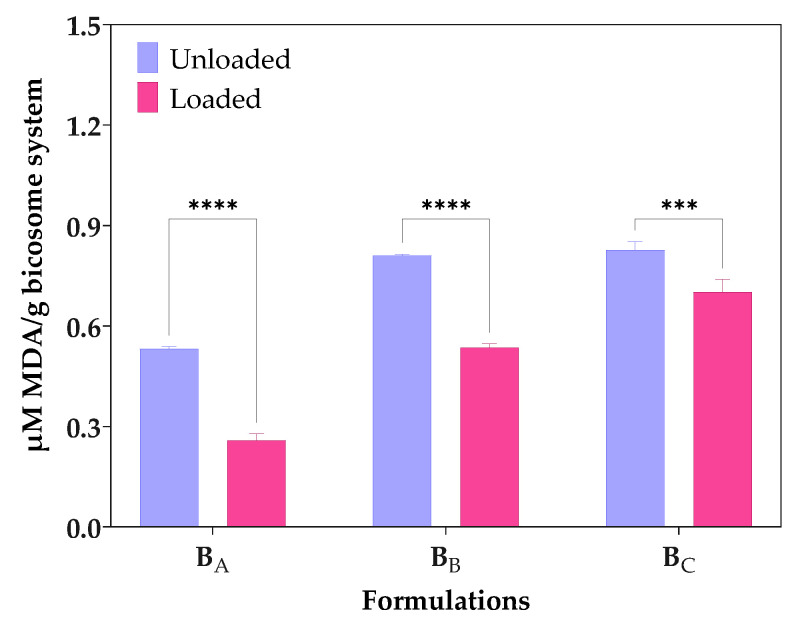
Lipid oxidation expressed as concentrations of malondialdehyde (µM/g MDA) formed by the breakdown of fatty acids of bicosome (B_A_, B_B_, and B_C_) loaded and unloaded with cur/α-toc. Statistical analyses of two-way ANOVA, Bonferroni, and multiple comparisons: **** = *p* < 0.0001, *** = *p* < 0.001 (*n* = 3).

TBARS method measures the level of MDA formed by the breakdown of unsaturated fatty acids [31]. Figure 3 shows the MDA equivalent content (µM/g) due to autoxidation of unloaded bicosomes and cur/α-toc-bicosomes (B_A_, B_B_, and B_C_). The results showed that the TBARS values for cur/α-toc-bicosomes (B_A_, B_B_, and B_C_) were lower than those of unloaded bicosomes. The MDA equivalent content (µM/g) values in the unloaded bicosomes increased with the increase in the total lipid percentages in the formulation B_C_ > B_B_ > B_A_, which reached 0.828 ± 0.03, 0.810 ± 0.01, and 0.532 ± 0.01 MDA equivalent content (µM/g), respectively. The same occurred when the formulation of the cur/α-toc-bicosomes reached 0.702 ± 0.05, 0.536 ± 0.02, and 0.258 ± 0.03 MDA equivalent content (µM/g) for B_C_, B_B_, and B_A_, respectively. In general, the presence of cur and α-toc reduces lipid oxidation. Bicosomes have the same concentration of cur and α-toc, but different lipid concentration; therefore, lipid oxidation increases with increasing lipid concentration. This is clearly reflected in the B_A_ formulation (lower lipid concentration), with the difference obtained in lipid oxidation between loaded and unloaded bicosome being 52%.

Lipid oxidation is a free radical chain mechanism, which can be described in terms of initiation, propagation, and termination processes. These processes can be complex because they include sequential and overlapping reactions [42]. Cur, α-toc, and cholesterol present in bicosomes have reported benefits in the oxidative stability of lipid formulations due to the phospholipid–hydrophilic molecule interaction [43,44]. Moreover, the intrinsic antioxidant capacity of cur and α-toc is able to protect bicosomes from free radicals, generating benefits in their oxidative stability, as shown in Figure 3.

Previous studies have reported that cur used at low-molar concentrations is monomeric and oriented along the membrane ordering the phospholipid bilayer. On the contrary, when cur is used at high-molar concentrations, it oligomerizes in the membrane, which reduces the thickness and alters the molecular order of the phospholipid bilayer [45]. In this study, the bicelles and bicosomes were loaded with a low concentration of cur, which could generate a monomeric orientation in the phospholipid bilayer. In addition, cur exhibits low solubility in water, but when it is encapsulated in bicelles and bicosomes, its solubility in water increases, which generates an increase in its availability to interact with free radicals and its capacity to donate hydrogen atoms to free radicals [46].

On the other hand, α-toc could also contribute to decreasing lipid membrane peroxidation in bicosome by acting in conjunction with cur since α-toc provides protection against the early events of lipid oxidation at the radical initiation site. Studies have established that oxidation is accelerated in liposomes without α-toc and is slower in α-toc-loaded liposomes [47].

### 3.3. Antioxidant Effectiveness Tests

**Figure 4:**
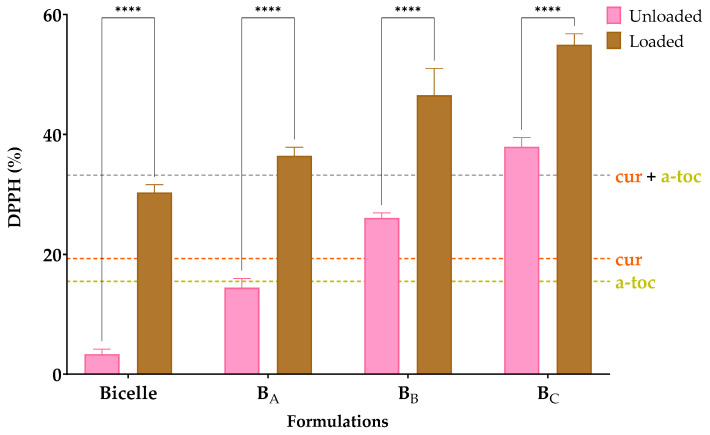
Antioxidative activity expressed as reduction of DPPH (%) radicals of bicelles and bicosomes (B_A_, B_B_, and B_C_) loaded and unloaded with cur/α-toc. Statistical analyses of two-way ANOVA, Bonferroni, and multiple comparisons: **** = *p* < 0.0001 (*n* = 3).

To evaluate the antioxidant effect of the cur and α-toc, a DPPH assay was performed. DPPH is a stable free radical that can be used to measure the radical scavenging activity of antioxidants. The reduction of the DPPH radical by radical scavengers is evaluated spectrophotometrically by monitoring the decrease in absorbance at 517 nm, as the DPPH radical is decolorized from deep violet to pale yellow [48]. The test was performed by comparing unloaded bicelles, cur/α-toc-bicelles, unloaded bicosomes, and the cur/α-toc-bicosomes (B_A_, B_B_, and B_C_). In addition, the equivalent concentration of the cur, α-toc, and cur and α-toc mixture used in the formulations was evaluated. Figure 4 shows the results of the antioxidant effect.

The DPPH values (%) achieved by unloaded bicelles and cur/α-toc-bicelles were 3 and 30%, respectively, while for unloaded bicosomes, the values achieved were between 14 and 38%, and for cur/α-toc-bicosomes, the values achieved were between 30 and 55%. The cur/α-toc-bicosomes recorded an increase in antioxidant effect of 89% compared to unloaded bicelles, while in the cur/α-toc-bicosomes, the greatest difference was presented by the B_A_ formulation, reaching a 60% increase in antioxidant activity compared to the unloaded B_A_ formulation.

Cur and α-toc do not lose antioxidant activity when encapsulated in bicelles and bicosomes; moreover, the antioxidant activity is higher with the increase in phosphatidylcholine in the composition of the bicosomes (B_C_ > B_B_ > B_A_), since as noted, the unloaded bicosomes present a significant antioxidant activity. This could be due to saturated fatty acids such as palmitic and stearic acid present in phosphatidylcholine (Lipoid P-100) used in the preparation of the bicosomes. These fatty acids have low sensitivity to oxygen and are not easily oxidized [39]; thus, the antioxidant capacity of the cur/α-toc-bicosomes is enhanced over the antioxidant capacity of free cur, free α-toc, and free cur and α-toc mixture.

On the other hand, the incorporation of cur, α-toc, and cholesterol can reduce the fluidity of the liposomal membrane or increase its hydrophobicity, and thus greatly enhance the activation energy required for polar small molecules to cross the liposomal membrane [49]. These effects are beneficial for reducing the oxidative damage and particle swelling of phospholipidic particles by inhibiting oxygen penetration and water penetration, respectively. Additionally, a study conducted by Chaves et al. [40] established that the co-encapsulation of cur and cholecalciferol in nanoliposomal vesicles causes a synergistic effect that increases antioxidant activity in the formulation, which could occur between cur and α-toc in our bicosomes.

### 3.4. Hemolysis and Cell Viability Assays

The hemolytic and cell viability evaluation of delivery systems is becoming an important issue nowadays, especially referring to lipid systems intended for pharmaceutical industrial application. A hemolysis test can be used as an index of hemocompatibility for materials intended for biological applications. None of the cur/α-toc-bicosomes (B_A_, B_B_, and B_C_) caused hemolysis (Table 4). According to ISO 10993-4, materials showing hemolysis values lower than 5% can be used as blood-contacting materials [50]. Thus, these results suggest that cur/α-toc-bicosomes had a good biosecurity and that hemocompatibility was not compromised by formulation components.

**Figure 5:**
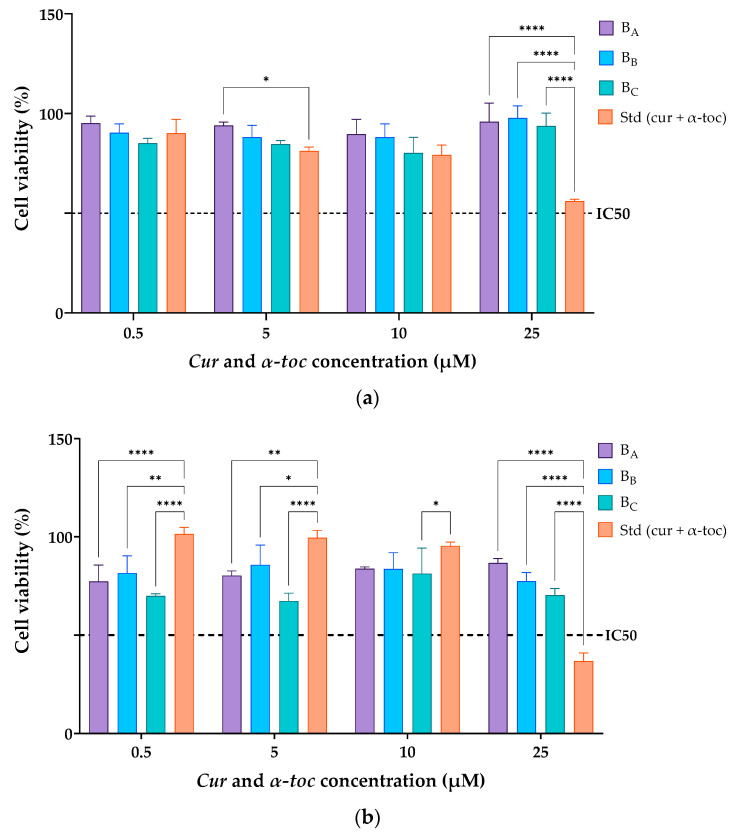
(**a**) Dermal fibroblasts (3T3L1/CL-173™) and (**b**) human keratinocyte (HaCaT) cell viability (%) after incubation with free cur/α-toc solution or cur/α-toc-bicosomes (B_A_, B_B_, and B_C_) for 24 h. Statistical analyses of two-way ANOVA, Dunnett, and multiple comparisons: **** = *p* < 0.0001, ** = *p* < 0.01, and * = *p* < 0.05 (*n* = 3).

Figure 5 shows the cell viability profiles of cur/α-toc-bicosomes (B_A_, B_B_, and B_C_) and free cur and α-toc solution in contact with fibroblast (3T3L1/CL-173™) and keratinocyte (HaCaT) cells in an MTT assay after 24 h. Different concentrations of cur and α-toc in the range of 0.5–25 µM were evaluated. The results revealed that the viability of fibroblast cells (3T3L1/CL-173™) was above 85% in the cur/α-toc-bicosomes (B_A_, B_B_, and B_C_) in the range of concentrations at 0.5–25 µM used for the study (Figure 5a). A decrease in cell viability was observed at 25 µM concentration of cur and α-toc free below 60%. The cur/α-toc-bicosomes had higher cell viability than cur and α-toc free, which may be from the capability of bicosomes to enhance the bioavailability of cur and α-toc inside the cells.

In keratinocyte cells (HaCaT), the results revealed that the viability was ≥65% in the cur/α-toc-bicosomes used (B_A_, B_B_, and B_C_) in the range of concentrations at 0.5–25 µM used for the study (Figure 5b). Keratinocyte cells are more sensitive to contact with cur/α-toc-bicosomes (B_A_, B_B_, and B_C_) than to contact with free cur and α-toc at concentrations of 0.5, 5, and 10 µM; however, a significant decrease in cell viability (%) is observed using free cur and α-toc at concentrations of 25 µM, reaching only 34% cell viability.

Since statistically significant differences (*p* > 0.05) were detected among the bicosomes at the same dose. It can be concluded that the total lipid percentage (12, 16, and 20% *w*/*v*) used to prepare the cur/α-toc-bicosomes had a negative impact on the biocompatibility of the formulations in fibroblast and keratinocyte cells. Additionally, for the cur/α-toc-bicosomes, the pattern of cell viability behavior (%) is the same in all concentrations, ordered as follows: B_A_ > B_B_ > B_C_. On the other hand, for fibroblast and keratinocyte cells, the IC50 values for free cur and α-toc and cur/α-toc-bicosomes are greater than 25 μM, except for free cur and α-toc, where the IC50 value is between 10 and 25 μM in keratinocyte cells.

Our results are comparable to those of Lundvig et al. [51], who reported an apoptotic response in HaCaT cells induced by high doses of cur (≥30 µM). Caddeo et al. [5] demonstrated that α-toc-loaded transfersomes (vesicles prepared with phosphatidylcholine and different polysorbates) showed no toxic effect in HaCaT cells; however, a dose-dependent decrease in viability (from ~95% to 75%) was observed in fibroblast cells. Several studies support the topical application of cur and α-toc-loaded delivery systems for wound healing. In addition, in vivo and ex vivo studies using α-toc-loaded nanoemulsions confirm the proliferative effect both in keratinocyte cells, fibroblast cells, and in human skin biopsies [52]. The interactions between keratinocyte and fibroblast cells are crucial for the proper execution of the wound repair phase [51]. Our results are a starting point for future ex vivo skin studies using cur/α-toc-bicosomes, which could have potential application in the treatment of wounds or skin and/or mucosal ulcerations based on formulation characteristics.

### 3.5. In Vitro Antifungal Effectiveness

**Figure 6:**
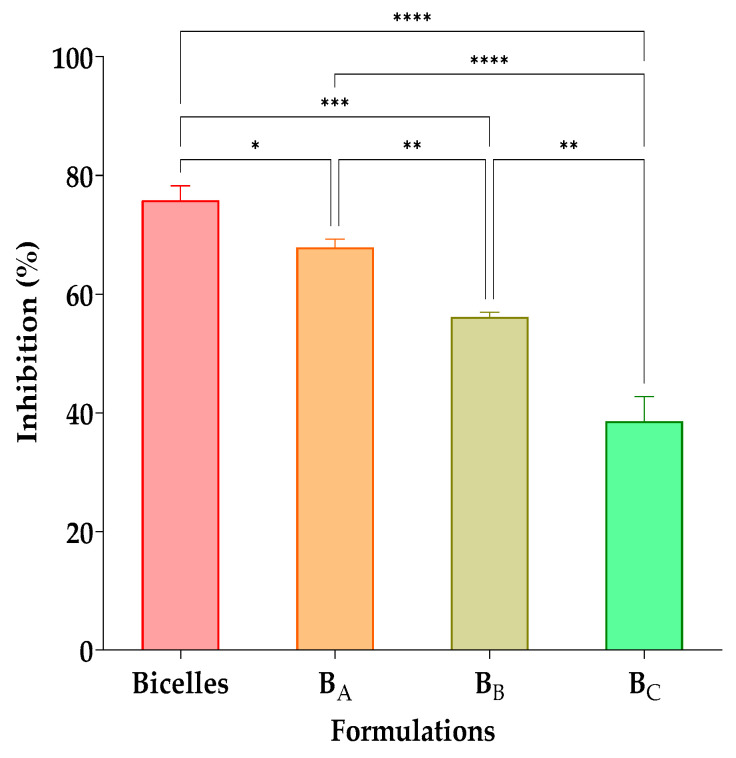
Antifungal effects of cur/α-toc-bicelles and cur/α-toc-bicosomes (B_A_, B_B_, and B_C_) against *C. albicans*. Different letters mean statistically significant differences. Statistical analyses of one-way ANOVA, Tukey, and multiple comparisons: **** = *p* < 0.0001, *** = *p* < 0.001, ** = *p* < 0.01, and * = *p* < 0.1 (*n* = 3).

The results of the in vitro antifungal activity on *C. albicans* of the cur/α-toc-bicelles and cur/α-toc-bicosomes (B_A_, B_B_, and B_C_) at a ratio of 50:50 *v*/*v* (inoculum:formulation) are shown in Figure 6. The highest antifungal activity (76 ± 2%) was observed in cur/α-toc-bicelles at a 50:50 *v*/*v* ratio. On the other hand, when increasing the total lipid percentage in the cur/α-toc-bicosomes (B_A_, B_B_, and B_C_), the growth of *C. albicans* also increases, reducing the antifungal activity. Inhibition values obtained were 68 ± 1%, 52 ± 6%, and 33 ± 10% for the B_A_, B_B_, and B_C_ formulations, respectively; this may occur because the liposomal vesicle surrounding the bicelles in the B_A_, B_B_, and B_C_ formulations makes the antioxidant molecules loaded in the systems less available. The B_C_ formulation has the highest lipid concentration in its composition (20% total lipid), which could make the interaction between cur and *C. albicans* less accessible. The antifungal effect of both free and encapsulated cur in different systems against *C. albicans* has been reported by several authors [53,54,55]. Shahzad et al. [56] established that the inhibition of *C. albicans* in the presence of cur is due to the generation of reactive oxygen species (ROS) toxic to the fungus and suppression of hyphal development. On the other hand, Xie et al. [57] substantiated that the result of encapsulation produces a gradual release of cur that destroys the cell membrane of *C. albicans*, increasing permeability, which generates a change in the internal environment of the fungus, thereby inhibiting its growth.

Both cur/α-toc-bicelles and cur/α-toc-bicosomes (B_A_, B_B_, and B_C_) are shown to reduce the growth of *C. albicans* in a 50:50 *v*/*v* ratio (inoculum:formulation); however, further studies are needed to demonstrate that our cur/α-toc-bicosomes can be used to treat diseases produced, for example, in mucosal tissue by the presence of *C. albicans*.

## 4. Conclusions

In the present study, we successfully prepared cur/α-toc-bicelles using DPPC, DHPC, cur, and α-toc. These bicelles were then encapsulated within liposomal vesicles composed of phosphatidylcholine (Lipoid P-100) and cholesterol, resulting in the formulation known as cur/toc-bicosome. Cur/α-toc-bicosomes represent a novel co-encapsulation approach that combines the inherent characteristics of liposomes with the versatility and applicability of bicelles, resulting in a unique and multifunctional nanostructure. Our results indicate that during the first step of encapsulation, bicelles significantly enhance the solubility of cur and α-toc when incorporated between the phospholipid bilayers. Furthermore, all prepared bicosomes exhibited high-entrapment efficiency for cur (56–77%) and α-toc (51–65%), varying based on the percentage of total lipids incorporated in the formulation. Moreover, cur/α-toc-bicosomes exhibited reduced lipid oxidation and increased antioxidant activity compared to unloaded bicosomes, suggesting a synergistic effect between cur, α-toc, and the phospholipids employed. Additionally, the formulations demonstrated good hemocompatibility, favorable biocompatibility in fibroblast and keratinocyte cell lines, and exhibited efficacy in inhibiting the growth of *C. albicans*. These findings suggest that the synergistic combination of two delivery systems (bicelles and liposomes) and two antioxidant compounds (cur and α-toc) could be effective systems for treating dermal diseases resulting from oxidative damage, such as psoriasis and ulcerations of the oral cavity caused by radiotherapy treatments. In addition, the external liposome protects the bicelle against dilution conditions, as occurs in environments with high water content. Therefore, the developed system may serve as a suitable delivery system for administration routes such as oral transmucosal. On the other hand, the potential capacity of cur/α-toc bicosomes to counteract dermal infections caused by *C. albicans* expands and diversifies their utility towards antifungal treatment, wound healing, cosmetics, and skincare. Future biological studies on cur/α-toc-bicosomes could explore various aspects, including in vitro release kinetics, ex vivo permeation and distribution in different tissues (such as skin and mucosal tissue), and the efficacy of the formulation through in vivo models.

## Figures and Tables

**Table 1:** Summary of the composition to prepare each cur/α-toc-bicosomes (B_A_, B_B_, and B_C_).

Bicosome System	DHPC/DPPC Relación Molar (*q*)	Cur (µM)	α-toc (µM)	Lipoid P-100/chol Ratio	Total Bicelle Lipid Concentration (% *w*/*v*)	Total Liposome Lipid Concentration (% *w*/*v*)	Total Bicosome System Lipid Concentration (% *w*/*v*)
**B_A_**	3.5:1	180	600	8:2	6	6	12
**B_B_**						10	16
**B_C_**						14	20

**Table 2:** Particle size (nm), volume (%), and polydispersity index (PDI) of unloaded bicelles and cur/α-toc-bicelles. Each value represents the mean ± standard deviation of at least three replicates. Different letters mean statistically significant differences with a *p*-value < 0.05.

Bicelles	Particle Size (nm)	Volume (%)	Polydispersity Index (PDI)
**Unloaded**	15 ± 0.1 ^a^	99 ± 1 ^a^	0.26 ± 0.07 ^a^
**Loaded**	16 ± 1 ^a^	99 ± 1 ^a^	0.28 ± 0.02 ^a^

**Table 3:** Particle size (nm) and volume (%) of two peaks for cur/α-toc-bicosomes (B_A_, B_B_, and B_C_). Each value represents the mean ± standard deviation of at least three replicates. Different letters mean statistically significant differences with a *p*-value < 0.05.

Bicosome Systems	Peak 1		Peak 2	
	Particle Size (nm)	Volume (%)	Particle Size (nm)	Volume (%)
**B_A_**	31 ± 0.5 ^b^	63 ± 14 ^a^	420 ± 7 ^a^	20 ± 9 ^a^
**B_B_**	55 ± 5 ^a^	57 ± 5 ^a^	388 ± 6 ^a^	38 ± 7 ^a^
**B_C_**	60 ± 8 ^a^	69 ± 4 ^a^	303 ± 1 ^b^	27 ± 1 ^a^

**Table 4:** Encapsulation efficiency (EE), loading capacity (LC), and hemolysis of the cur/α-toc-bicosomes (B_A_, B_B_, and B_C_). Each value represents the mean ± standard deviation of at least three replicates. Different letters mean statistically significant differences with a *p*-value < 0.05.

Bicosome Systems	EE cur (%)	EE α-toc (%)	LC cur/α-toc (%)	Hemolysis (%)
**B_A_**	56 ± 5 ^b^	58 ± 8 ^b^	52 ± 0.4 ^b^	ND
**B_B_**	69 ± 4 ^a^	64 ± 2 ^a^	65 ± 2 ^a^	ND
**B_C_**	77 ± 2 ^a^	65 ± 3 ^a^	67 ± 2 ^a^	ND

ND: not detected.

## Data Availability

Not applicable.

## Supplementary Materials

The following supporting information can be downloaded at: https://www.mdpi.com/article/10.3390/pharmaceutics15071912/s1, Figure S1: Histograms of the particle size distribution (nm) by intensity (%) and volume (%) for cur/α-toc-bicelles and cur/α-toc-bicosomes; Figure S2: Physical stability of cur/α-toc-bicosome B_C_ formulation determined by particle size (nm) initial and after 90 days storage at 4 °C; Table S1: Particle size (nm) and intensity (%) of two peaks for cur/α-toc-bicosomes (B_A_, B_B_, and B_C_).
