# Supplementary material for: Co-Encapsulation of Curcumin and α-Tocopherol in Bicosome Systems: Physicochemical Properties and Biological Activity
Source: Pharmaceutics. 2023 Jul 9;15(7):1912. doi: 10.3390/pharmaceutics15071912 (PMC10383532; doi:10.3390/pharmaceutics15071912)
Supplement: Supplementary file 1 [file pharmaceutics-15-01912-s001.zip › pharmaceutics-2444071-supplementary.pdf]

Supplementary Material

Co-encapsulation of curcumin and α-tocopherol in bicosome systems: physicochemical properties and biological activity

Daniela Vergara [1,*], Olga López [2], Claudia Sanhueza [1], Catalina Chávez-Aravena [3], José Villagra [3], Mariela Bustamante [4], Francisca Acevedo [1]

[1] Center of Excellence in Translational Medicine (CEMT), Faculty of Medicine, and Scientific and Technological Bioresource Nucleus (BIOREN), Universidad de La Frontera, Casilla 54-D, 4780000 Temuco, Chile
[2] Department of Chemical and Surfactant Technology, Institute of Advanced Chemistry of Catalonia (IQAC-CSIC), C/ Jordi Girona 18-26, 08034 Barcelona, Spain
[3] Laboratory of Pharmaceutical and Cosmetic Bioproducts, Center of Excellence in Translational Medicine (CEMT), Department of Preclinical Sciences, Faculty of Medicine, Universidad de La Frontera, Casilla 54-D, 4780000
[4] Temuco, Chile Center of Food Biotechnology and Bioseparations, Scientific and Technological Bioresource Nucleus BIOREN, Universidad de La Frontera, Casilla 54-D, 4780000 Temuco, Chile
* Correspondence: daniela.vergara@ufrontera.cl (D.V)

Table S1. Particle size (nm) and intensity (%) of two peaks for cur/α-toc-bicosomes (B_A B_B and B_C). Each value represents the mean ± standard deviation of at least 3 replicates. Different letters mean statistically significant differences with a p-value < 0.05.

Bicosome systems	Peak 1		Peak 2	
	**Particle size (nm)**	**Intensity (%)**	**Particle size (nm)**	**Intensity (%)**
$\mathbf{B_A}$	94 ± 7^{a}	$17 \pm 4^{a,b}$	411 ± 7^{a}	$74 \pm 5^{a,b}$
$\mathbf{B_B}$	67 ± 11^{a}	26 ± 1^{a}	327 ± 6^{b}	74 ± 1^{b}
$\mathbf{B_C}$	43 ± 3^{b}	19 ± 1^{b}	283 ± 17^{c}	86 ± 1^{a}

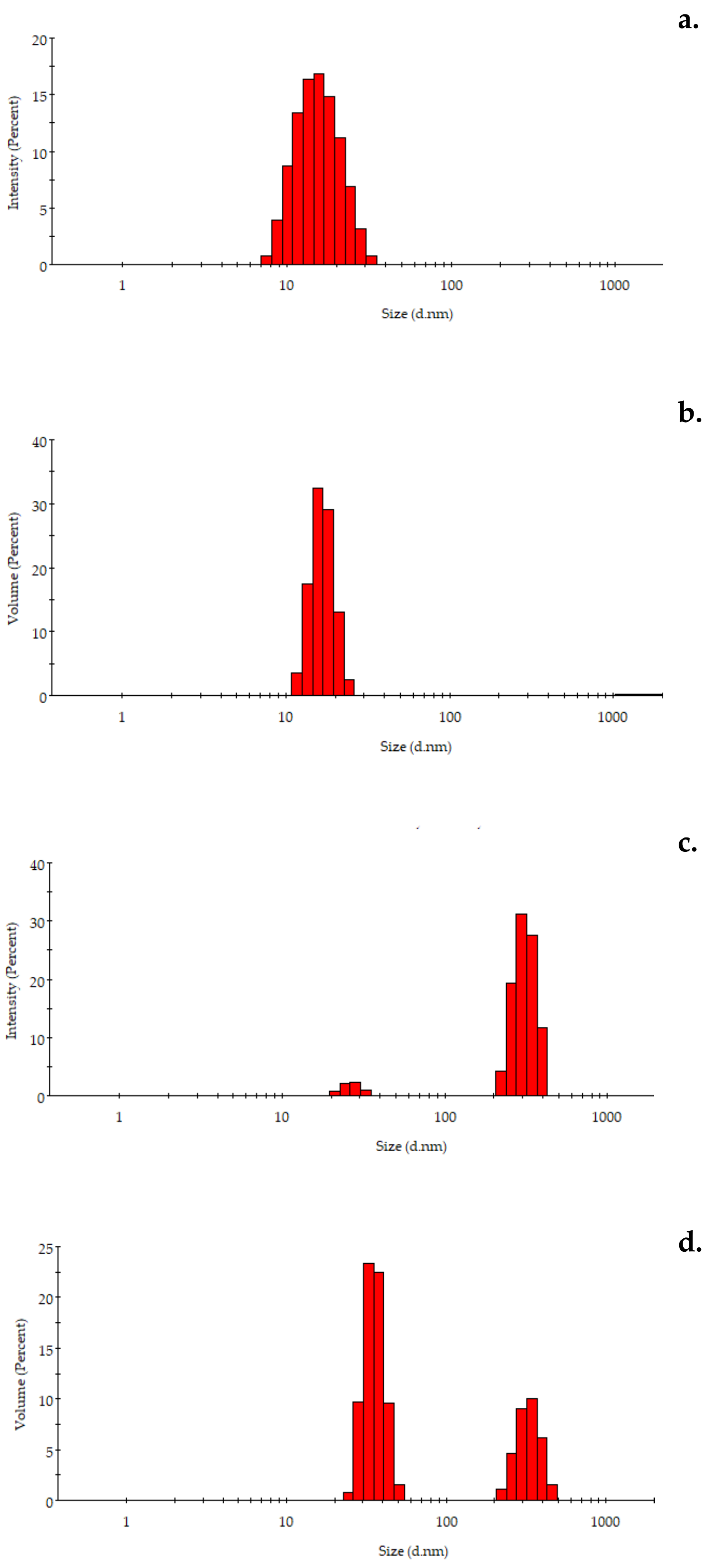

Figure S1. Histogram of the particle size distribution (nm). (a) cur/α-toc-bicelles by intensity (%), (b) cur/α-toc-bicelles by volume (%), (c) cur/α-toc-bicosome by intensity (%), and (d) cur/α-toc-bicosome by volume (%).

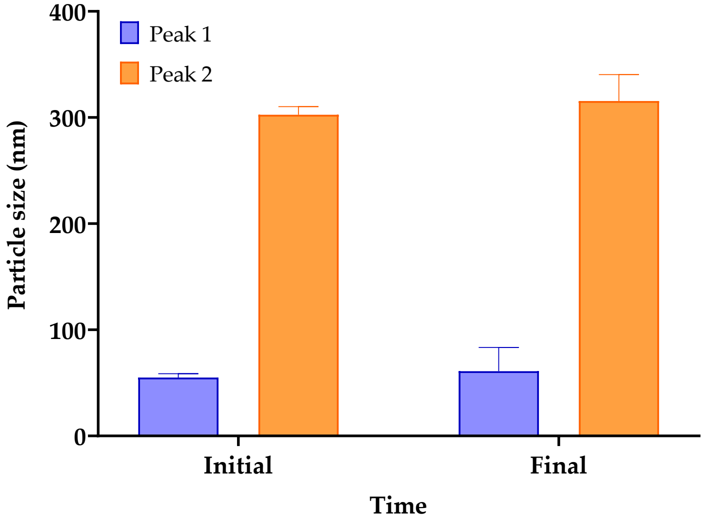

Figure S2. Physical stability of cur/α-toc-bicosome B_C formulation determined by particle size (nm) initial and after 90 days storage at 4 °C.
